# Facilitating access to primary care for people living in socio-economically vulnerable circumstances in Belgium through community health workers: towards a conceptual model

**DOI:** 10.1186/s12875-023-02214-2

**Published:** 2023-12-20

**Authors:** Caroline Masquillier, Theo Cosaert

**Affiliations:** 1 Department of Family Medicine and Population Health, Faculty of Medicine and Heath Sciences & Department of Sociology, Centre for Population, Family and Health, University of Antwerp, Sint-Jacobstraat 2, 2000 Antwerp, Belgium; 2 Department of Public Health, Institute of Tropical Medicine, Antwerp, Belgium

**Keywords:** Community health workers, Access to primary care, People living in socio-economically vulnerable circumstances, Conceptual model, Primary care, Outreach, Photovoice

## Abstract

**Introduction:**

Inspired by examples in low- and middle-income countries, 50 community health workers (CHWs) were introduced in Belgium to improve access to primary care for people living in socio-economically vulnerable circumstances. This article aims to explore the ways in which CHWs support people living in socio-economically vulnerable circumstances in their access to primary care.

**Methods:**

The qualitative research focuses on the first year of implementation of this pioneer nationwide CHW programme in Belgium. To respond to the research aim, thirteen semi-structured in-depth interviews were held with people living in socio-economically vulnerable circumstances. In addition, a photovoice study was conducted with fifteen CHWs comprising four phases: (1) photovoice training; (2) participatory observation with each CHW individually; (3) an individual semi-structured in-depth interview; and (4) three focus group discussions. The transcripts and the observation notes were analysed in accordance with the abductive analysis procedures described by Timmermans and Tavory.

**Results:**

The qualitative results show that the CHWs’ outreaching way of working allows them to reach people living at the crossroads of different vulnerabilities that are intertwined and reinforce each other. They experience complex care needs, while at the same time they face several barriers that interrupt the continuum of access to primary care – as conceptualised in the theoretical access-to-care framework of (Levesque et al. Int J Equity Health. 12:18, 2013). Building on the theoretical access-to-care framework described by (Levesque et al. Int J Equity Health. 12:18, 2013), the conceptual model outlines first the underlying mechanisms of CHW-facilitated access to primary care: (I) outreaching and pro-active way of working; (II) building trust; (III) providing unbiased support and guidance in a culturally sensitive manner; and (IV) tailoring the CHWs’ approach to the unique interplay of barriers at the individual and health system level along the access-to-care continuum as experienced by the individual. Further disentangling how CHWs provide support to the barriers in access to care across the continuum and at each step is outlined further in the process characteristics of this conceptual model. Furthermore, the qualitative results show that the way in which CHWs support people is also impacted by the broader health system, such as long waiting times and unwelcoming healthcare professionals after referral from a CHW.

**Discussion:**

The conceptual model of CHW-facilitated access to primary care developed in this article explores the way in which CHWs support people living in socio-economically vulnerable circumstances in their access to primary care in Belgium. Through their outreaching method, they play a valuable bridging role between the Belgian healthcare system and people living in socio-economically vulnerable circumstances.

**Supplementary Information:**

The online version contains supplementary material available at 10.1186/s12875-023-02214-2.

## Highlights

- Community health workers (CHWs) reach people who live at the crossroads of different vulnerabilities.

- CHWs adjust their support to the unique interplay of barriers at the individual and health system level across the access-to-care continuum.

- Through their outreaching method, they play a valuable bridging role between the Belgian healthcare system and people living in socio-economically vulnerable circumstances.

- The conceptual model of CHW-facilitated access to primary care explores the ways in which CHWs support people living in socio-economically vulnerable circumstances in their access to primary care in Belgium.

**Supplementary Information:**

The online version contains supplementary material available at 10.1186/s12875-023-02214-2.

## Introduction

Access to timely, affordable and acceptable healthcare is a fundamental human right [1]. However, inequity in access to healthcare persists in all countries around the world – inhibiting Sustainable Development Goal 3, which aims to ensure healthy lives and promote well-being for all at any age [2]. International literature has affirmed the inverse care law, ‘which states that those with the greatest health needs receive the least healthcare services’ ([3]: p. 130). Studies show that people living in vulnerable conditions have a higher risk of being in poorer health and therefore have higher care needs [4, 5]. In addition, for people living in socio-economically vulnerable circumstances, access to healthcare poses an important and growing challenge in certain countries, including Belgium [3, 6–8].

Belgium is below the European average with regard to equal access to healthcare [9]. The group of people experiencing difficulties in accessing Belgian healthcare is growing. In 2008, 1.4% of the lowest income population indicated that they did not have access to the necessary care; in 2016, the gap increased to 8% [7]. In line with international findings, people living in socio-economically vulnerable circumstances in Belgium are primarily people with a low level of education and/or a low income [5, 7]. Due to a complex interplay of barriers, people living in socially vulnerable circumstances find it difficult to access healthcare, resulting in, among other things, the postponement or avoidance of care [5, 8].

People with limited or no access to health services are often described as ‘*hard to reach*’. But from their perspective, it is the health services that are hard to reach ([4] p. 25–26). In Belgium, several measures have already been implemented to make healthcare more affordable and more accessible [9]. However, the growing gap indicates that these measures are not sufficient [7]. Innovative ways are needed to make healthcare more accessible to these underserved vulnerable populations [10].

Low- and middle-income (LMICs) countries face similar challenges with access to healthcare for people living in socially vulnerable situations – albeit on a larger scale. For a large proportion of the population, these countries have shown creativity and developed innovative practices to ensure access to primary care in a context of limited resources [11, 12]. One of these innovations is the introduction of community health workers (CHWs) into the health system, whose history dates back to the Barefoot Doctors in China in the 1950s. Renewed attention for CHWs was sparked with the Alma-Ata Declaration as a way to strengthen primary healthcare [13–15]. CHWs are trusted members of local communities, who have shared life experience with the people they support. They have received limited training to, for example, help people living in socio-economically vulnerable circumstances navigate the healthcare system. They bring care closer to patients, their families and communities [16, 17]. The literature shows that CHWs in LMICs can support the strengthening and accessibility of primary healthcare – making progress to ensure equitable access to quality and comprehensive healthcare [16, 18–21].

Inspired by examples in LMICs and building on decades of community-based care experience with diseases (such as tuberculosis) in their own regions [22], there is growing interest in employing CHWs in high-income countries, especially the English-speaking countries [23–25] – such as the United States [16, 26], Australia [25] and the United Kingdom [27]. Academic literature highlights that CHWs can play an important role in directing people to healthcare, providing culturally appropriate care, health education and advocacy – resulting in reduced health inequalities and positive health outcomes for people living in socially vulnerable conditions [28–32]. The COVID-19 pandemic highlighted the importance of CHWs worldwide and was a catalyst for the introduction of the first federal CHW programme in Belgium to improve access to primary care for people living in socio-economically vulnerable circumstances [33]. At the end of 2020, the Belgian Federal Government gave the National Institute for Health and Disability Insurance and the National Intermutualistic College the task to employ the first CHWs throughout the country to improve access to primary care. In ten cities, socio-economically vulnerable neighbourhoods were selected, where a total of 50 CHWs were deployed. This qualitative research focuses on this pioneer nationwide CHW programme in Belgium.

Conceptual frameworks have been developed to uncover the way in which CHWs facilitate patients’ adoption of healthy behaviours [34], mediate health gains in clients [35], and provide access to care in clinical settings for patients with a history of high acute care usage or uncontrolled conditions [36]. However, so far there is limited research available to support the development of a broader conceptual model, which explores the outreaching way of working used by CHWs to facilitate access to primary care for people living in socio-economically vulnerable circumstances. Such a conceptual model is vital to guide and facilitate an optimal deployment of CHWs and to enhance their impact. Building on the work of previous scholars, a conceptual model in this article is depicted as a “diagrammatic form of a conceptual framework which is refined as data collection and analysis takes place” ([37]: p. 36) and “immediately applicable to a particular study” ([38]: p. 189). Responding to this research gap, this article aims to explore the ways in which CHWs support the access to primary care of people living in socio-economically vulnerable circumstances in Belgium and consequently develop a conceptual model of CHW-facilitated access to primary care.

## Theoretical framework

**Fig. 1:**
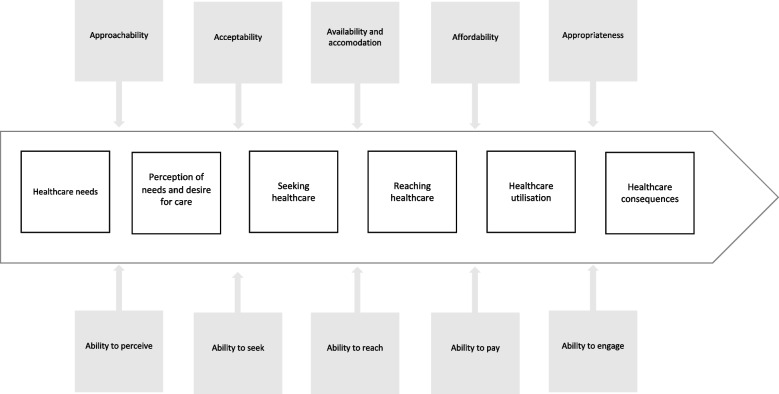
Access-to-care continuum (adapted from work by Levesque et al. [39])

‘Access to healthcare’ is a complex concept that has been conceptualised in several ways in academic literature [39]. In this article, we adopt the view of access to primary care as a continuum, as developed by Levesque and colleagues [39], which has been used widely and successfully [40]. Levesque and colleagues define access to healthcare as a continuum that starts with having a health need, after which the authors describe five more stages: (1) the perception of the health and care need; (2) the possibility to find the right care; (3) reaching the right care in a timely manner; (4) being able to use this care appropriately; and (5) having the actual health need fulfilled by these healthcare services (*cf. Middle section, *Fig. 1) [39].

Health services are not always designed and organised to reach and assist underserved vulnerable populations [4, 10]. In this respect, the authors identify organisational barriers related to the accessibility of healthcare services (*cf. Upper part, *Fig. 1*)* [39]. Previous research has shown the distribution of primary care resources is unequally distributed in urban settings, with poor geographic accessibility to primary healthcare services in socio-economically disadvantaged neighbourhoods [41]. In addition to barriers at the organisational level, Levesque and colleagues identify barriers at the individual level inhibiting access to care. To successfully navigate the access-to-care continuum, an individual needs certain abilities (*cf. Lower section, *Fig. 1) [39]. The abilities of people living in socio-economically vulnerable circumstances may be inhibited by, for example, language barriers, social isolation, mobility challenges, precarious job conditions and financial difficulties [8, 42]. Consequently, these people sometimes feel less able to cope with their health needs and find it more difficult to access healthcare [39, 42].

## Methods

In this article, we respond to our research aim by focusing on the results of in-depth interviews with people living in socio-economically vulnerable circumstances and a photovoice study with CHWs involved in the Federal Belgian CHW programme. This qualitative research was part of larger study, in which coaches were also involved in focus group discussions to explore the supportive structure that is needed to support CHWs with their tasks. However, these focus group discussions are beyond the scope of this article.

### Sampling recruitment strategy

The respondent groups were recruited differently: (1) CHWs and (2) people living in socially vulnerable conditions who receive CHW support. To recruit CHWs, all those involved in the Belgian CHW programme were invited to participate. The CHWs who showed interest received more information about the study. In order to make people living in socially vulnerable circumstances feel as comfortable as possible with participating in the study, CHWs participating in the photovoice study were invited to introduce the study to the people they supported. The CHWs asked these people if they would be willing to participate in the study through a semi-structured in-depth interview. On the days that the researcher conducted participant observation activities following the CHWs on their daily activities, a brief visit was then scheduled with the people who had given permission through the CHW to participate in the study. Before starting the observation, the CHW first discussed with the person they were supporting whether they felt comfortable meeting the researcher. If the researcher was welcome, the purpose of the study, the design, and aspects such as voluntariness and confidentiality were explained to the participant in an understandable way. Furthermore, during this meeting between the researcher and the individual living in socio-economically vulnerable circumstances, the study and a time to conduct the in-depth interview were agreed upon. These steps were intended to create a basis of trust and thus leave room for a well-considered decision to participate or not. When the actual interviews took place with the people living in socio-economically vulnerable circumstances, the CHWs were not present.

### Sample size

Five CHWs participated per region with a total of fifteen CHWs across all three regions in Belgium. In Flanders, there were five female CHWs aged between 30 and late 50s. In Brussels, two women and three men accepted the invitation to participate in the study. Their ages ranged from late 20s to early 40s. In Wallonia and the German-speaking community, four men and one woman participated, aged between mid-20s to mid-30s.

A total of thirteen people living in socio-economically vulnerable conditions – six men and seven women – participated in the semi-structured in-depth interviews. They were aged between 20 to 85 and all live at intersections of different circumstances that cause them to live in socio-economically vulnerable circumstances, including but not limited to: uncertainties regarding residency status; poverty; and migration background.

### Data collection

Thirteen semi-structured in-depth interviews were conducted by one of the authors (TC) with people living in socially vulnerable conditions who received support from a CHW until data saturation was reached. The interviews were conducted between April and September 2021. Topics discussed during these interviews included (cf. Supplementary file 1: Interview guides): the access-to-care continuum and the barriers they experienced; history and current use of health services; current care needs; knowledge of health services; and their experience of the CHW support they received.

In addition, a photovoice study was conducted by one of the authors (TC) in which the fifteen CHWs participated between April and September 2021. Photovoice is a ‘community-based participatory research method’, in which the respondents are active participants in the research [43]. Respondents take pictures related to the research questions. These photos are used as conversation stimuli in in-depth interviews and focus group discussions (cf. Supplementary file 1: Interview guides). The photovoice study comprised four phases. In the first phase, a photovoice training was organised per region, which included a detailed explanation of the research process, the use of the camera, the use of photography in research, and ethics in research and photography [43, 44]. The CHWs were then given three weeks to take photographs related to the research questions. After about two weeks, the second phase started. In the second phase, participatory observation was conducted with each CHW individually to follow them during their daily tasks. During this field visit, the researcher spent a day with each CHW as a participant observer and took structured fieldnotes. Here, the researcher assumed the role of ‘*observer-as-participant*’: the researcher ‘is known and recognized, but only related to the respondents of study, as a researcher’ ([45]: p. 54). Afterwards, the researcher discussed how the photo-taking went, refreshed the research questions and answered any questions the CHWs still had regarding the research method. In the third phase, a semi-structured in-depth interview was conducted with each CHW individually. In this in-depth interview, the photos were used as visual stimuli for discussion to obtain more information about what the pictures depicted and why they were taken [46]. During the semi-structured in-depth interviews, the photographs were personally discussed with each CHW according to the photovoice SHOWED technique, which structured the conversation and prompted discussion [47] (cf. Supplementary file 1: Interview guides). The method was participatory in the sense that the respondents chose which pictures they wanted to take and which pictures they wanted to discuss as a priority during the in-depth interviews. Furthermore, the CHWs provided their own analysis of the images during the interview [47]. The fourth phase comprised of a concluding focus group discussion in each region with the five CHWs. In these three focus group discussions, the five CHWs presented their selection of photos to each other, discussed the research questions and shared their own experiences and views with the other CHWs from the same region.

After receiving written informed consent, the semi-structured in-depth interviews and the focus group discussions were recorded with an audio recorder. Interviews and focus group discussions were conducted in Dutch, French or a language of the respondent’s choice with the support of professional translators who had experience interviewing people living in socially vulnerable conditions. The translators involved signed a confidentiality agreement. Three people living in socially vulnerable circumstances did not wish the in-depth interview to be recorded for fear that this might affect their residence permit. Instead, the researcher took detailed notes. The interviews and focus group discussions for which permission was obtained to make an audio recording were subsequently transcribed and pseudonymised. The semi-structured interviews lasted one hour on average, while the focus group discussions lasted two and a half hours on average.

### Analytical strategy

The transcripts and the observation notes were imported into NVivo (version 1.2) for analysis. The transcripts provide a detailed representation of the respondents’ answers. The transcripts were conducted by a professional external organisation – who signed a processing agreement in order to comply with the General Data Protection Regulation (EU GDPR) guidelines. For two respondents, the transcription was also translated from Arabic into English. For the semi-structured interviews with people living in socio-economically vulnerable circumstances for which permission was not obtained to use audio recording, the detailed notes taking during the interview were imported into NVivo.

Data collection and reading of the transcripts was alternated to further inform and evaluate subsequent in-depth interviews and focus group discussions when saturation was reached. The photographs were not analysed by the researcher as data: only the descriptions the CHWs gave during the in-depth interviews and focus group discussions were analysed. The data was analysed by carefully reading and rereading the transcripts as well as the field notes. The data was first coded openly, then axially and finally selectively [48]. During this phase of coding, memos were also made. A sub-sample of the transcripts was also coded by another researcher in order to compare and discuss similarities and differences with a view to intercoder reliability [49]. The analysis was carried out in accordance with the abductive analysis procedures described by Timmermans and Tavory [50] – where is built on theory as sensitizing notions when analysing empirical data and where theory and analysis are constantly interwoven [51].

After the data analysis was completed, two feedback sessions were held, to which all CHWs participating in the study were invited for member checking. Due to COVID-19 measures, these feedback sessions were organised online per language (one in Dutch; one in French). During these sessions, the results were presented. After this presentation, feedback was requested from the CHWs, certain themes were further explored together, and expertise was exchanged between the CHWs. This member check from the CHWs was incorporated into the results.

It is important to reflect on our position as researchers. The authors of this report are highly educated, white, middle-class researchers. The respondents only knew us as researchers. In this context, one could describe our role as that of the ‘stranger’, an outsider [52]. The advantage of this position is that it created a safe place for respondents to share their experiences. However, this can also have disadvantages, such as the possibility of a different interpretation of what was said and meant by the respondents. The interpretation of the data and the writing out of the results can be coloured by the perspective we have from our social position. We tried to limit misinterpretation as much as possible through meticulous data analysis and the feedback sessions on the results with the CHWs who participated in the study.

## Results

Below we explore the ways in which CHWs support people through their outreaching way of working. After describing the care needs of people living in socio-economically vulnerable circumstances in this study, we discuss the ways in which CHWs support people living in socio-economically vulnerable circumstances to overcome barriers to accessing primary healthcare.

### Outreaching way of working

Various outreach strategies were used by the CHWs who participated in this study. A first outreach strategy involves being present in public spaces, where one can reach a wide audience. For instance, by organising activities in a park with games for children, music, free drinks and food to attract people and introduce the Federal Belgian CHW programme, or by organising an information stand at a local market. A second strategy used by CHWs is to focus specifically on places where people living in socio-economically vulnerable circumstances are often present, such as: going door-to-door in social housing blocks; being present in a social restaurant, at a food bank or in a homeless centre. A third strategy to reach people living in socio-economically vulnerable circumstances is to work closely with local organisations. These organisations can be of a non-medical nature, for instance, a local organisation for newly-arrived female migrants. CHWs may also develop close collaborations with healthcare services, such as an emergency department of a local hospital or they leave flyers or business cards at local pharmacists. A fourth strategy used by CHWs is to be present on the streets in collaboration with other organisations and people working in the community, such as a community guard or street worker. Finally, CHWs also make publicity in different ways to reach people living in socio-economically vulnerable circumstances in an indirect way. This can be on paper, such as using flyers about the Federal Belgian CHW programme in different languages, or digitally by being present on social media platforms.

*On the street, we always have to adapt. Sometimes we are allowed inside, sometimes outside. It was nice weather and quite by chance, we passed by a resident, and she had a question. So we were actually allowed [to enjoy] a coffee offered on the street. And that actually gave me the feeling of I should take a picture here of how approachable and vulnerable we make ourselves, because we go to fellow human beings and we adapt to those people.*

*CHW, Flemish region*

### Care needs of people living in socio-economically vulnerable circumstances

The Federal Belgian CHW programme reached a diverse group of people living at the crossroads of different vulnerabilities that are intertwined and reinforce each other. More specifically, it concerns the following potential vulnerabilities: having a low level of education; being at risk of poverty and severe material deprivation; having no or limited knowledge of the local language; being of an older age; having a limited social network; being a newcomer; having no legal residence status or being in request for international protection; having a physical disability; living with a mental illness; and/or being homeless.

The diversity of the people reached by CHWs, who each live at a crossroads of different vulnerabilities, results in a wide variety of and often a combination of different care needs. At the time of this study, these needs were related to COVID-19-related care, such as COVID-19 testing and vaccination, and primary care. With regard to primary care needs, these mainly included diagnosis and follow-up of physical complaints, such as pain, and finding a general practitioner (GP). The CHWs also indicated that there is a high demand for dental care, especially among people without a residence permit. Although the CHWs mainly help with needs related to physical care, there also appears to be a prominent need for mental healthcare. Topics such as depression, trauma and chronic stress regularly emerged during the in-depth interviews. Needs were not only directly linked to healthcare, but also encompassed broader needs regarding living circumstances and well-being, mainly related to habitable housing and the need for a legal residence permit. These needs can have a strong impact on a person’s mental and physical health.

*The only problem is with housing, and that’s the problem with people who get sick. [...] If you for example don’t find a place to live, you will get very stressed. You will get so stressed until you get sick. You will have to sleep in the street and you will fall sick. So you will sleep in the street and your situation won’t get better. So you will fall sick little by little with pressure and all, and that’s what happens to people, and then people start to become aggressive.*

*Person living in socio-economically vulnerable circumstances, Brussels region*

### How CHWs support people living in vulnerable circumstances to overcome barriers to access to primary care

Results show that the social position of a person at the crossroads of different vulnerabilities leads to specific care needs and a complex interplay of barriers with regard to the fulfilment of their care needs. Depending on the intersection of vulnerabilities that an individual finds themselves at, specific barriers across the access-to-care continuum occur together. These barriers cause people to experience difficulties in accessing care, which can lead to their care needs being exacerbated. CHWs indicated that some care needs and barriers can be reduced to a simple question, to which a CHW can offer a relatively quick answer. Often, however, a person experiences a complex interplay of care needs and barriers that require CHW support over a longer period of time as discussed by the CHWs. Depending on an individual’s specific interplay of barriers and their needs, the support can be one-off or long-term.

As set out in the theoretical framework, we look at barriers across the access-to-care continuum [39]. In this section, we distinguish between overarching barriers that affect the entire access-to-care continuum and barriers that are linked to a specific phase in the access-to-care continuum.

#### Overarching support provided by CHWs along the access-to-care continuum

People experience overarching barriers along the entire continuum, such as: lack of trust; administrative burden and digital divide; language barrier; and legal status.

##### Lack of trust

The interviews with the people living in socio-economically vulnerable circumstances and the CHWs show that a previous negative experience with care personnel, such as poor quality of service provision, discrimination or the feeling of not being taken seriously, can damage someone’s confidence in the healthcare sector, hence increasing the threshold to seek help again. Furthermore, qualitative results from interviews with both groups show that people living in socio-economically vulnerable circumstances sometimes feel unheard or not understood. This perceived lack of understanding and trust has an impact on all steps of the access-to-care continuum. A CHW listening to their experiences and stories is usually the first step towards a bond of trust. This bond of trust is an essential basis from which a CHW can start to tackle other barriers. However, building trust takes time. The CHWs indicated that at the beginning of some cases they also have to provide support for needs that are not directly related to healthcare, such as food and shelter, in order not to damage the fragile trust relationship. In addition to creating a bond of trust, the CHW also has a motivating and activating function, with the aim of supporting people living in socio-economically vulnerable circumstances to autonomously overcome barriers in the future. For instance, various CHWs indicated that they show their clients how to make a digital appointment the first time, or go along to a healthcare provider as a confidant once, but after that they mainly encourage them to take these steps independently.

*A few days later [name CHW] phoned me, you need help. Yes, I need help, I can’t manage with my papers, I’m always on my own. That’s when [name CHW] started coming once a week. I’m very happy that he comes and that I have someone now. If I have a problem or things aren’t going well today, now I know there’s someone who’ll listen to me and that makes me feel so much better. […] I can talk, I used to have a lot of weight here and now I know I have someone to talk to, to tell him my problems, to tell him that I need this, all that helps me a lot.*

*Person living in socio-economically vulnerable circumstances, Wallonia region and German-speaking community*

##### Administrative burden

The interviews with CHWs and the people living in socio-economically vulnerable circumstances show that the administration and paperwork surrounding the care system is also an important barrier. Almost all people who were supported by a CHW expressed in the interview that they experience this barrier and it occurs at almost every step of the access-to-care continuum. The complex and difficult language of the associated administration means that people do not always understand what rights they can invoke, what is expected of them, or who they should address to in order to receive certain care or support. This can result in people not receiving the care and support they are entitled to on time. The photovoice study with the CHW shows that the CHWs help with collecting the necessary documents or by explaining complex terms and administrative procedures, for example. They also often refer the person to the relevant services for specific paperwork, such as the social services of the health insurance. Mostly CHWs will also provide follow-up to ensure the administrative hurdles have been overcome.

*Also concerning health, if I receive a paper or a bill at home that I don’t understand, I take a picture and send it to him [CHW], then he tells me what’s going on. [...] Yes, anything concerning health, anything [...] And he goes with me to the lawyer.*

*Person living in socio-economically vulnerable circumstances, Brussels region*

##### Digital divide

Not everyone has a digital device or the necessary skills to navigate the health system digitally. Digitalisation has further increased under the pressure of the COVID-19 epidemic, making access to primary care more difficult for certain groups, such as the elderly and non-native speakers. The CHWs offer support in various ways, for example, by helping to make an online appointment with a doctor or by requesting documents via the online administration of a health insurance company. The CHW shows on a device of the person in question what to do, or the CHW uses his or her own device.

##### Language barriers

In several interviews with people living in socio-economically vulnerable circumstances as well as CHWs, the language barrier between a person in need of care and the healthcare provider came up as an important barrier along the entire continuum of access to primary care. This problem arises not only during consultations, but also in the administration of the care system or when trying to obtain information. The CHW supports people in their communication with health services along all steps of the access-to-care continuum. This may involve both communicating a problem from an individual to a healthcare provider and translating information from a healthcare provider to an individual. If the CHW speaks the language in question, some CHWs themselves act as interpreters – even though this is not strictly part of their tasks. In other cases they manage with gestures, drawing symbols, using translation applications, or they put the people living in socio-economically vulnerable circumstances in contact with an interpreter who then supports them further, possibly in cooperation with the CHW. CHWs noted that they can also provide support to people who speak the local languages but have difficulties with medical jargon or complex language used in administrative documents.

*That's it, and there's nothing in the facilities to facilitate this communication. In most facilities they ask them to bring an interpreter with them, some of them don't know anyone, so they bring a child. The doctor opposite talks for fifteen minutes, the child says two words or a sentence, and we're not even sure that the information has been passed on correctly.*

*CHW, Wallonia Region and German-speaking community*

##### Legal status

The qualitative results from interviews with both groups show that an important group of people living in socio-economically vulnerable circumstances supported by CHWs are people without legal residence status. Whether or not a person has a legal residence permit determines to a large extent which rights they have regarding healthcare. The absence of a legal residence permit can therefore create an additional legal barrier along all the steps of the access-to-care continuum. Interviews with CHWs and people living in socio-economically vulnerable circumstances show that CHWs will first try to make sure that the person in question obtains access to reimbursement for healthcare costs through social services. In addition, the CHWs refer people to organisations that specifically work with people living in socio-economically vulnerable circumstances, such as Doctors of the World and Médecins Sans Frontières. In the interviews with the CHWs, however, it became clear that people without a legal residence permit are often suspicious of social services due to previous negative experiences.

#### Support that CHWs provide to overcome barriers specific to steps in the access-to-care continuum

In addition to the overarching barriers, the qualitative results show that people experience specific barriers to each step of the access-to-care continuum, as presented by Levesque and colleagues [39].

##### The perception of needs and desire for care

**Fig. 2:**
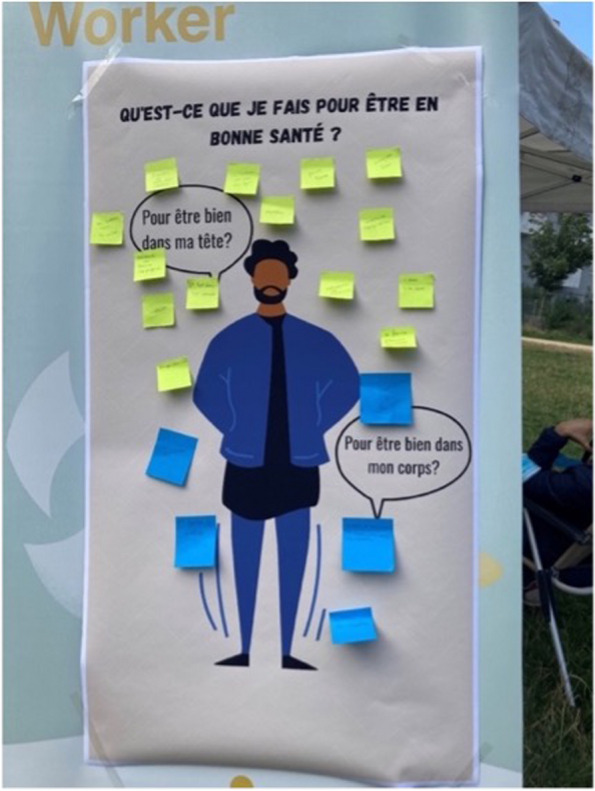
Generating discussion about the perception of needs and desire for care

The interviews with the CHWs show that people living in socio-economically vulnerable circumstances do not always immediately express their care needs. According to the interviewed CHWs, this can have various causes, such as distrusting or being suspicious of government services, or experiencing difficulties in identifying or articulating their own care needs. By taking the time and creating a bond of trust, the CHW is usually able to identify ‘*the question behind the question*’* (*CHW, Flanders region*)* (cf. Fig. 2).

*The man is really basic, it’s really the human being, a human body and all that. And the idea is really to give clients the choice of sticking post-it notes depending on where it hurts, where they feel pain on the man and then depending on what they’ve identified as aches and pains, we can come back at the end to propose a new appointment, to discuss in detail what you’re going to do about this ailment you’ve identified, have you met a doctor, do you need a doctor, etc. That’s it! The idea is really to generate discussion.*

*Two CHWs, Brussels region*

CHWs indicated in the photovoice study that it is not always easy for people to determine what information about health and the health system is correct and what is not. They indicated that inadequate or misinformation can cause people to fear and/or distrust healthcare and healthcare providers. In addition, CHWs pointed out that fear, distrust and misinformation can cause people to have a false perception of the need for care, to be reluctant to seek care, or to experience doubts about the usefulness of, for example, vaccination. One of the ways in which CHWs support people living in socio-economically vulnerable circumstances is by providing them with correct health information. CHWs try to engage in conversation and listen without judgment to people’s uncertainties and doubts. In addition, in cooperation with specialised organisations, workshops and information sessions are also given to groups on specific themes, such as emergency medical assistance, sexual health and digital skills.

##### Seeking healthcare

**Fig. 3:**
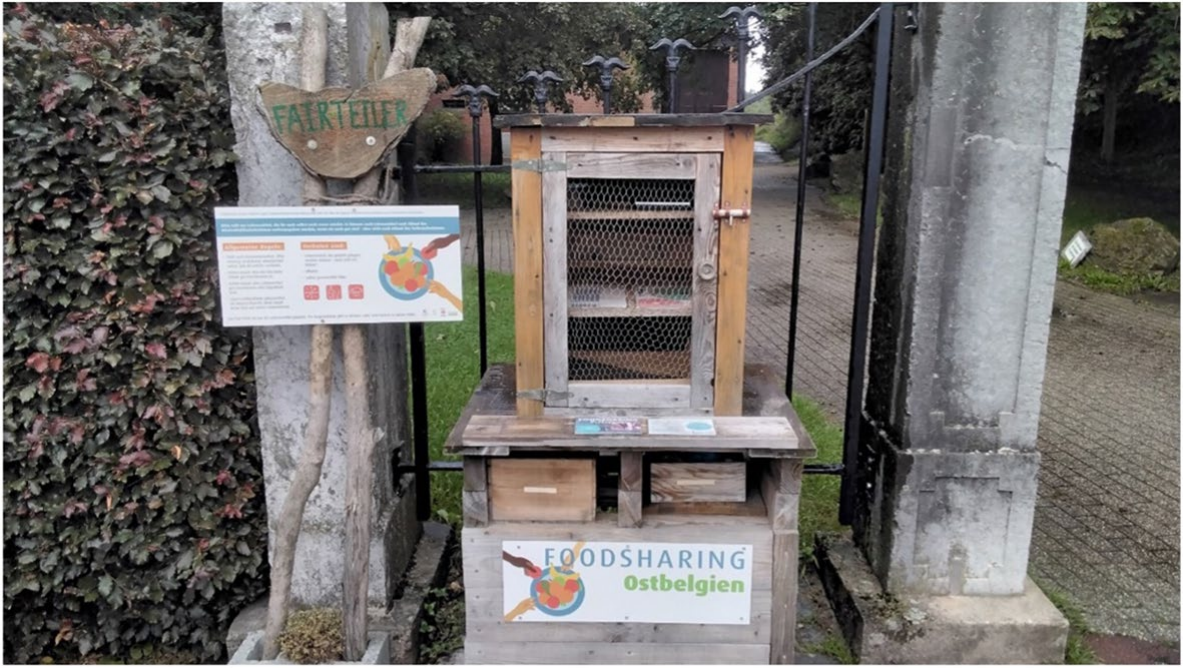
Supporting to navigate the healthcare system

The qualitative results show that people living in socio-economically vulnerable circumstances do not always know how to navigate the healthcare system. Interviews with the CHWs show, on the one hand, that this barrier arises because people are not aware of which care services and types of support exist. On the other hand, there is also a group of people who do know what services exist, but find it difficult to obtain more information or make contact. The latter can be caused both by a too limited range of services and by too many services with no clear overview. In the interviews with the CHWs, it is specified that this barrier mainly occurs among newly-arrived migrants who are not yet familiar with the Belgian healthcare system. Older people who are socially isolated also experience these challenges with regard to finding the right care, because they cannot fall back on their own social network for suggestions and advice. A central element of the work of CHWs is to support people living in socio-economically vulnerable circumstances in navigating the healthcare system (cf. Fig. 3). In concrete terms, they explain the different organisations in the healthcare landscape and refer them to the right actors. For instance, they help them to find a GP, a district health centre, or other specific services.

*I don’t know if you’ve heard of this little Food Sharing project? You can put down your food so that people who need it can eat it. And it’s empty. It’s always empty. But it’s a bit symbolic because it shows that people just don’t know that it exists. It’s really in a remote corner of [name region] and I think it’s just a lack of information for people. It’s a bit for the [healthcare] service too, it’s not used as it could be.*

*CHW, Wallonia region and German-speaking community*

A barrier that was frequently mentioned in the interviews with CHWs and individuals living in socio-economically vulnerable circumstances is the limited primary care offer in their neighbourhood. This usually concerns saturated district health centres or a shortage of GPs and dentists in a neighbourhood, resulting in patient stops or waiting lists. The CHWs try to find healthcare providers without waiting lists or patient stops, in order to help the person find timely care. If certain needs can be better met by welfare organisations that are not directly linked to health, CHWs direct them there. These organisations include, for instance, social services, a social housing organisation or a lawyer (e.g. to discuss someone’s residence status).

##### Reaching healthcare

**Fig. 4:**
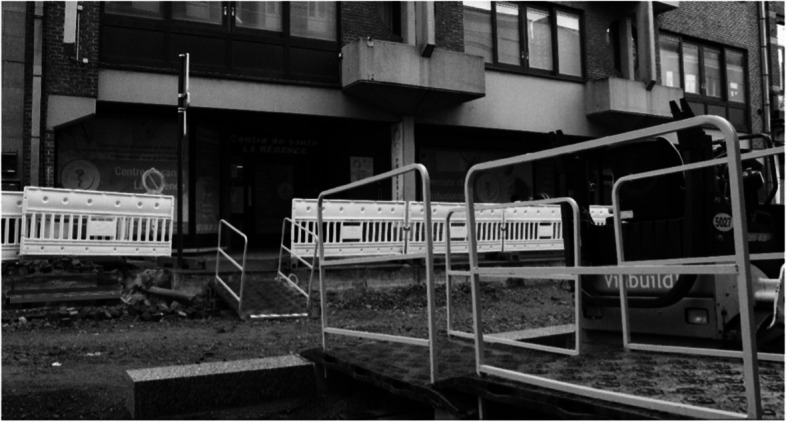
Barriers in reaching healthcare. Picture: CHW, Wallonia Region and German-speaking community

Experiencing difficulties in reaching health services is a literal barrier to healthcare access. The qualitative results from interviews with CHWs and people living in socio-economically vulnerable circumstances show that not everyone has the means to reach a healthcare provider independently. Especially for people with limited mobility, alternative modes of transport are often essential, but sometimes these are inaccessible, unknown or unaffordable. Lack of mobility goes hand in hand with the fact that the geographical location of healthcare services can also be a barrier (cf. Fig. 4). A CHW looks for the most appropriate support, based on a specific need, for instance by exploring possibilities for public transport to overcome barriers to physical movement. Results show that, if needed, CHWs will sometimes accompany the individual in question to reassure and motivate them or show them the route.

*Sometimes, an isolated person simply does not know how to get to the bus and what to do to get to the hospital, etc. So, what I do, I can accompany them a first, a second, a third time to get them to confide in me a little, to see how and what I should do with the bus, and I can also put them in contact with the transport organisations that exist.*

*CHW, Wallonia Region and German-speaking Community*

For people living in socio-economically vulnerable circumstances who have a precarious job, making an appointment is not always possible, for instance, when they have to stay on the phone for a long time before they can make an appointment with the health insurance provider or the social services. If it is not possible to take a day off at work for the consultation, the CHW will look for a way to make an appointment after working hours.

##### Healthcare utilisation

Interviews with both CHWs and people in socio-economically vulnerable circumstances show that if people have found the way to the right healthcare provider, they may still also experience barriers during the consultation. For example, the cost of consultations in primary care poses a problem, especially for people who do not have a valid residence permit and only have limited rights with a medical card. An important note to be made here is that in some cases people are entitled to reimbursement but are not aware of it. When a person experiences a financial barrier to making use of certain healthcare, CHWs help in various ways. For example, there are CHWs that help to obtain reimbursement from the health insurance provider or financial support from the social services. In addition, a CHW can also refer to support in case of debts after unexpected high medical expenses. In order to facilitate urgent situations, there are also CHWs who, in exceptional cases, advance medical expenses such as hospitalisation or medication and then reclaim them from the relevant authorities. This was mentioned by both CHWs and people living in socio-economically vulnerable circumstances.

*Some do, they are afraid or something. Some go to the dentist, mostly they are afraid and they don’t want to make an appointment, but I explain it to them: it would be better to make an appointment once a year, then you would pay less. With me they do. That’s why I join them to the dentist.*

*CHW, Flanders region*

In some situations, the CHW will accompany the person to visit health services, such as the GP or the health insurance offices. Depending on the barrier or barriers the person experiences, this support can be provided for various reasons: help with communication in case of a language barrier; physical support in case of reduced mobility; or support in case of low confidence in care services. Regarding language barriers, the CHWs support with communication between patient and healthcare provider goes beyond the need for translation. In their interviews, several CHWs mention that, even if they speak the same language, it is not always easy for both parties to make themselves understood. The CHW helps in such situations by explaining exactly what one can ask of or expect from a healthcare provider. With regard to trust, it is especially important when individuals have little confidence in healthcare providers to join for a first visit. In a conversation between the healthcare provider, the patient and the CHW, the CHW may or may not be present during the consultation or may wait in the waiting room and talk to the healthcare provider before and/or after the consultation. Both the people living in socio-economically vulnerable circumstances and CHWs indicated that involved services and healthcare providers are sometimes more accessible and helpful when they are accompanied by a CHW, and that the care request of the person in question is taken more seriously. Their presence can therefore also have a facilitating effect in that respect.

##### Healthcare consequences

From the interviews with the CHWs, it became clear that people sometimes struggle with how to follow the advice of a healthcare professional for various reasons, for example: because the doctor uses technical jargon; or the doctor speaks a language different to the one of the patient; or because the patient experiences fear and distrust. These reasons sometimes render it difficult for people living in socio-economically vulnerable circumstances to ask additional questions. On the one hand, this can cause problems in understanding and interpreting, for example, the results at a GP. On the other hand, in such situations it may be unclear to the person in question what is expected of them or what the next steps are. This can form a challenge when they receive a referral to another healthcare provider. CHWs also help to interpret and explain results when they are complex or not completely clear. The CHWs indicated that they almost always contact the people living in socio-economically vulnerable circumstances after an appointment to hear to how it went. The CHW can then follow up to see if next steps need to be taken. The interviews with the CHWs show that people do not always know what to do with the healthcare provider’s advice. For example, when the person in question has received a referral to a specialist or another service. From the interviews with the people living in socio-economically vulnerable circumstances it appears that this follow-up is highly appreciated and that they feel well supported by it.

*Sometimes I call to get results. Some people don’t understand the doctor, the results, so they sit with me and we call the doctor together to learn about the results or to make an appointment.*

*CHW, Flanders region*

## Discussion

This article aims to explore the ways in which CHWs support people living in socio-economically vulnerable circumstances in their access to primary care in Belgium by developing a conceptual model of CHW-facilitated access to primary care – which will be outlined in this discussion. The qualitative results show that CHWs can reach people living at the crossroads of different vulnerabilities that are intertwined and reinforce each other – an idea rooted in the concept of intersectionality [53, 54]. In addition, the results indicate that these people experience complex care needs on the one hand, and various barriers at the health system and individual level to their access to primary care, on the other. The results of this study underscore the idea that CHWs can play a valuable bridging role between people living in socially vulnerable circumstances who have little to no access to regular healthcare and the Belgian healthcare system. The unique position CHWs occupy between the community and the health system constitutes an essential advantage of their functioning, but this position requires that CHWs have both trustful relationships and credibility within their communities and a functional relationship with actors in the health system [55–58].

### Towards a conceptual model of CHW-facilitated access to primary care

**Fig. 5:**
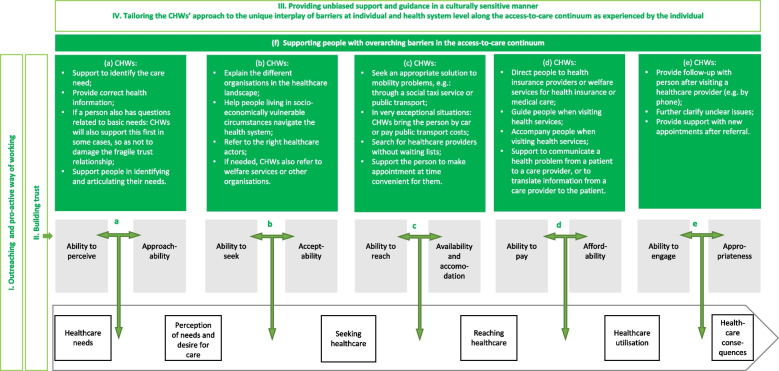
Conceptual model of CHW-facilitated access to primary care

The conceptual model presented in Fig. 5 builds on the theoretical access-to-care framework described by Levesque and colleagues [39].The conceptual model outlines first the underlying mechanisms of CHW-facilitated access to primary care (cf. Fig. 5, I-IV): (I) outreaching and pro-active way of working; (II) building trust; (III) providing unbiased support and guidance in a culturally sensitive manner; and (IV) tailoring the CHWs’ approach to the unique interplay of barriers at the individual and health system level along the access-to-care continuum as experienced by the individual. Further disentangling how CHWs provide support to the barriers in access to care at each step (cf. Fig. 5a-e) and across the continuum (cf. Fig. 5 f) is outlined in the process characteristics.

However, important to note is that the qualitative results show that the way in which CHWs support people is also impacted by the broader health system, such as: legal regulations around reimbursement for people without legal residence; long waiting times; unwelcoming healthcare professionals after referral from CHWs; and division of powers in Belgium between federal and regional levels, and human resource shortages at primary care centres, among others. These are significant barriers on the supply side, on which CHWs have very limited impact. Whereas CHW have to operate within the limits of the broader health system when supporting an individual person, they do signal these structural barriers to policy makers and other stakeholders involved.

### Underlying mechanisms

The qualitative results show that a core underlying mechanism of the CHW’s approach is their outreaching and pro-active way of working, allowing them to reach people living in socio-economically vulnerable circumstances (cf. Fig. 5, I). Building trust is a key step (cf. Fig. 5, II) before CHWs can provide unbiased support and guidance in a culturally sensitive manner (cf. Fig. 5, III). Through their pro-active way of working, they help underserved people living in socio-economically vulnerable circumstances along the access-to-care continuum and tailor their approach to the unique interplay between health system barriers and individual barriers experienced by people living in socio-economically vulnerable circumstances (cf. Fig. 5, IV) along the access-to-care continuum – as described by Levesque and colleagues [39].

- I. Outreaching and pro-active way of working
  The CHWs’ outreaching way of working is an important strength of their approach (cf. Fig. 5, I), as also underscored in international literature [57]. They do this in different ways, for instance: by being present in public spaces; by focusing specifically on places where people living in socio-economically vulnerable circumstances are often present; by working closely with both welfare and healthcare organisations; or by being present at other organisations. CHWs use a pro-active approach to visit people living in socio-economically vulnerable circumstances at home or in their neighbourhood, where these people feel most comfortable. The outreaching way of working removes the barrier to taking the first step towards healthcare. As such, they are able to offer accessible and low-threshold support.
- II. Building trust
  The qualitative research results show that people living in socio-economically vulnerable circumstances often experience a lack of trust in care and healthcare providers, which can be triggered by previous negative experiences – which is in line with previous research [59]. Qualitative results indicate that this mistrust forms a barrier along the entire access-to-care continuum. Results show that CHWs have the time to build a relationship of trust, which forms an important basis from which CHW can start to address other barriers (cf. Fig. 5, II) – as found in international literature [60–62]. Previous research shows that because CHWs have a shared background with the people they support, they have more relevant cultural competences to provide support and this recognition also facilitates trust building [59, 61]. CHWs use strategies such as empathic communication and perseverance – derived from indigenous knowledge – to support people in their health (Pinto et al., 2012). Previous research indicates that in addition to recognition between CHWs and the people they support, equality and reciprocity further facilitates this trustful relationship [59]. The qualitative results show that building trust, however, takes time and is fragile. For example, the CHWs in the photovoice study indicated that they also support people with welfare questions so as not to damage this incipient bond of trust. Furthermore, the qualitative results show that it is important for CHWs to be able to adopt an independent stance and not be linked to certain organisations to avoid associating themselves with organisations that have a negative reputation among people living in socio-economically vulnerable circumstances. Furthermore, if an individual has a negative experience with a healthcare provider after referral from a CHW, results show that this can be detrimental to the trust they have in the CHW’s services – in line with international research findings [62].
- III. Providing unbiased support and guidance in a culturally sensitive manner
  Limited knowledge of the local language is an important barrier that is reflected in every step of the access-to-care continuum. This language barrier is also highlighted as a significant barrier to access to primary care in other European countries [63]. Qualitative results show that language support can be important both for people who speak the local language and for non-native speakers. For both groups, jargon and/or the complexity of the healthcare provider’s message may result in the person living in socio-economically vulnerable circumstances not being able to process the information. In such situations, the CHW will not only help to translate communication between the healthcare provider and patient, but also to support people in their communication along the continuum of accessing care. For example, when formulating the actual care request (cf. Fig. 5a); when communicating a health problem from a patient to a healthcare provider and providing contextual background if needed (cf. Fig. 5d); or when conveying information from a healthcare provider to the patient (cf. Fig. 5d and e).
  Their shared life experience, such as migration background, socio-economic status, living in the same neighbourhood or knowing it well, also enables them to offer culturally sensitive support (cf. Fig. 5, III). The literature supports this and states that this goes beyond overcoming a language barrier, but also includes understanding the social conditions in which people live and local knowledge and attitudes [25] and the local knowledge and attitudes towards health [61]. This unique understanding of the experiences, language, culture and socio-economic reality of the people they support contributes to the success of CHWs [64]. This shared lived experience makes it more likely that they will better understand the needs and barriers of people living in socio-economically vulnerable circumstances and that community acceptance will be greater [61].
- IV. Tailoring the CHWs’ approach to the unique interplay of barriers at individual and health system level along the access-to-care continuum as experienced by the individual
  CHWs can make an important contribution to bringing people living in socio-economically vulnerable circumstances into mainstream primary care in a way that is tailored to their individual needs and the barriers they experience. Some people have specific needs to which the CHW can offer answers in the short term. Others experience a combination of different barriers to successfully navigating the health system. For these more complex requests for help, a longer follow-up by the CHW is often desirable. Depending on the care needs and interplay of the individual and health system barriers experienced, CHWs support people along the entire access-to-care continuum (cf. Fig. 5, IV).
  A recent scoping review of patient navigation interventions in low- and middle-income countries found no intervention addressing all individual abilities along the access-to-care continuum [65]. The CHWs in the Federal Belgian CHW programme take the first steps in this direction. Because the CHWs tailor their support to each individual and are able to provide long-term support and follow-up, they can help address the various barriers experienced along the access-to-care continuum.

### Process characteristics

CHWs provide support in this study at different steps along the access-to-care continuum (cf. Fig. 5a-e), for instance, but not limited to, by: supporting people to identify their care needs (cf. Fig. 5a); helping people living in socio-economically vulnerable circumstances to successfully navigate the fragmented health and welfare offerings (cf. Fig. 5b); trying to find an appropriate solution when mobility is a barrier to care (cf. Fig. 5c); accompanying people when they visit health services and if needed raising awareness during an interaction with care provider about the (cultural) vision people have about health and healthcare (cf. Fig. 5d); contacting the person after an appointment to hear how the consultation went, and then seeing together if the CHW can provide further support, for example, by making an appointment with a specialist (cf. Fig. 5e). Both the interviews with the people living in socio-economically vulnerable circumstances and with the CHWs showed that services involved are more accessible and helpful when the CHW is also present.

In addition to the support at each step of the continuum, CHWs also provide support – building on a basis of trust – for barriers experienced throughout the entire access-to-care continuum, such as administrative support and overcoming the digital divide (cf. Fig. 5, f). The qualitative results show that people living in socio-economically vulnerable circumstances experience difficulties in identifying the rights to which they can appeal – in line with a recent report by Doctors Of the World [63]. The qualitative results show that in almost all steps of the access-to-care continuum, CHWs try to help with clarifying the rights people are entitled to and to support with administrative matters. Furthermore, the CHWs in this study help people overcome the digital divide, which has been exacerbated by the COVID-19 pandemic where more services opted for digital solutions [66]. In doing so, they can help close the gap ‘between those who are quick to catch up and those who risk being left behind by technology literacy [and] health literacy’ ([67]: p. 40). Qualitative research shows that CHWs support people without legal residence by trying to obtain the possibility for reimbursement for this person through the social services and by referring them to organisations which are specialised in providing support for this group of people.

### Limitations and strengths

In order to be able to interpret the research results correctly, it is important to indicate the limitations of this study. First, there is a possible bias in the selection of respondents, namely CHWs and individuals living in socio-economically vulnerable circumstances. The self-selection among CHWs may lead to a selection bias. In addition, there is a possible bias in the selection of individuals living in socio-economically vulnerable circumstances, as the CHWs approached potential interviewees first in order to not damage trust. Second, the number of interviews with individuals living in socio-economically vulnerable circumstances was limited, so these findings should be interpreted with caution. A thorough examination of the various barriers experienced by people living in socially vulnerable conditions over time is beyond the scope of this study. Further research is therefore needed to map out in-depth the complex interplay of barriers over time along the access-to-care continuum in cooperation with people living in socio-economically vulnerable circumstances. Third, it is important to note that the COVID-19 pandemic and related measures are likely to have further aggravated some of the barriers, such as the digital divide and difficulties in navigating the healthcare system, due to the temporary closure of certain services and organisations. Fourth, the qualitative results do not allow us to make statements about the impact of the CHWs’ work, nor about their reach or the outcomes. In the future, it is important to explore the reach, impact and outcomes of the CHWs’ work through a mixed methods study*,* which combines both quantitative and qualitative longitudinal research. Fifth, a focus on the broader contextual factors influencing the Federal Belgian CHW programme, such as community dynamics, economic and political context, and health system, was beyond the scope of this research project. Further research is required to study the Federal Belgian CHW programme in this broader contextual framework [68], for example, by questioning how the Federal Belgian CHW programme can be connected to or embedded into the healthcare system is strongly recommended.

In addition to these limitations, we note some strengths of the study. First, different respondents – individuals living in socially vulnerable conditions and CHWs – were surveyed across all three regions of Belgium, resulting in nuanced results from different perspectives. Second, the participatory photovoice research process allowed for rich data collection as CHWs were engaged in the study at different points in time. Photovoice research provides a more insider perspective and insight into the work of the CHWs at different times. In this research method, the CHWs take on more of an expert role and are also ‘co-producers’ of knowledge. Research literature shows that respondents in this method also gain more self-confidence and self-esteem [43, 46, 47, 69]. Paying attention to the experience of the CHWs is seen as an important element for the structural improvement of CHW programmes [68]. Third, this qualitative research builds on the work of Levesque and colleagues [39], where accessibility is seen as a continuum in contrast to many studies where accessibility is only seen as ‘the use of care’ [5, 39]. To the best of our knowledge this is the first study to design a conceptual model on how CHWs improve access to primary care by building on the seminal work of Levesque and colleagues [39].

### Supplementary Information

**Additional file 1. **Interview guide.

## Data Availability

The qualitative pseudomised data is available upon reasonable request from the authors.
