# Supplementary material for: Facilitating access to primary care for people living in socio-economically vulnerable circumstances in Belgium through community health workers: towards a conceptual model
Source: BMC Prim Care. 2023 Dec 20;24:281. doi: 10.1186/s12875-023-02214-2 (PMC10731868; doi:10.1186/s12875-023-02214-2)
Supplement: Supplementary file 1 — Additional file 1. Interview guide. [file 12875_2023_2214_MOESM1_ESM.pdf]

<u>IN-DEPTH INTERVIEW (IDI) – SEMI-STRUCTURED INTERVIEW GUIDE CHWs</u>

PART 1: Getting to know the respondent

First, I would like to get to know you a little bit better. I would like to invite you to briefly introduce yourself.

(Probe socio-demographics: age, migration background, experience before being a CHW, experience as a CHW; ***educational background****)*

Thanks so much for your introductions.

Introductory questions:

- Where are you working?
- How many people have you helped so far?
- What group of people are you helping?
- What are the main challenges these people experience?
- Could you help them to overcome these challenges?
    - Which ones were successful?
    - Which ones were not?
- Could you help people to improve their access to primary care?
    - When could you help? And how?
    - When could you not help? Why?

PART 2: Discussing the photographs

1. **<u>Select the five best and most relevant photos (to the research question) of every photographer</u>**

   From the photographs taken, each participant will be asked to select and talk about the five photographs they feel are most significant. Following the questions formulated by (Hergenrather, Rhodes et al. 2009), the photographs are discussed. During this discussion various themes, such as success and challenges, will be discussed.

2. **<u>Find out the story behind each of those five photos</u>**

In this activity, the photographer is going to give background information on the five photos they have selected. Make sure it is clear why the participant took these photos

Questions that can be helpful (SHOWeD, modified):

1. Could you describe your picture? What do we see here?
2. What is really happening in your picture?
3. Why did you take a picture of this? Why do you want to share this?
4. What does the story behind this picture tell us about your work as CHW?
5. Why does this problem / concern / strength exist?
6. What can we do about it?

3. Write captions for every photo

Now that we have selected and discussed these five photos, I would like to ask you write a caption for all of them. A caption is a short piece of writing in which you explain what the picture means to you in a way that is understandable to the other CHWs in the focus group discussion.

PART 3: Focusing on themes relevant to the research question

Thank you for sharing all of this with me, I definitely learned a lot from it. Now I would like to focus a bit more on some of the themes that you already touched upon.

A. Difficulties and challenges
- What are the most difficult aspects of your job as a CHW?
- What could help you to overcome these challenges?
    - *Probe: CHW supervisor, other tools,….*
- If any, what unintended side-effects did you job have?

B. Successes
- What went really well so far in your job?
- What do you like about your job as a CHW?

C. Training
- How was the training you received?
    - *Probe: in what ways did this provide you with the necessary skills? In what ways not?*
- Did you receive refresher training?
- What could improve?

D. Tools
- What do you think about the tools you received to work with?
    - *Probe: take the tools and discuss*
- What is good?
- What should be changed?

E. Administration
- What do you think of the administration part of your job?
    - *Probe: workload, IT system,…*

F. Follow-up with supervisor
- How does the supervisor help you?
- Have you had the feeling you were supported and could share your worries, difficulties, challenges with the supervisor?
- What can be improved?

G. Incentives
- Do you feel like you are sufficiently reimbursed for the work that you do?
- Is the is valuable career experience for you?
    - *Probe: where do you see yourself after this?*

H. Future
- If the project could continue to exist. What should be kept the same?
- What should change?

- Do you have other ideas of what should be done differently?

PART 4: Concluding the IDI

Thank you for participating in this interview.

- What was it like to be a photovoice photographer?
- Are there any pictures you would not like to be displayed?
- Are there other things you would like to share?
- Do you have remarks regarding this interview? Do you have any questions for me?

Sources:

Hergenrather, K. C., S. D. Rhodes, C. A. Cowan, G. Bardhoshi and S. Pula (2009). "Photovoice as community-based participatory research: A qualitative review." American journal of health behavior **33**(6): 686-698.
Rutgers (2016). PhotoVoice: Facilitator's Guide. Rutgers, Rutgers**:** 132.

FOCUS GROUP DISCUSSION – TOPIC GUIDE CHWs

PART 1: Getting to know the respondents

We have all met each other before, during the photovoice workshop, but as a good reminder, I would like to invite you to briefly introduce yourself to the group.

(all participants introduce themselves)

Thanks so much for your introductions.
You are all very welcome.

PART 2: Presenting the photographs
Now I would like to invite you to present your selected photographs (that you chose during the in-depth interview) to your colleagues. Could you tell us what this photograph presents and what you would like to share related to your work as a CHW.

The researcher can prompt this discussion by quickly going over the SHOWeD structure.

PART 3: Discussing the research questions

Now as a short exercise, could you group your photos not by person, but by theme?

I. Difficulties and challenges
- What are the most difficult aspects of your job as a CHW?
- What could help you to overcome these challenges?
  - *Probe: CHW supervisor, other tools,….*
- If any, what unintended side-effects did you job have?

J. Successes
- What went really well so far in your job?
- What do you like about your job as a CHW?
- Who are you helping?

K. Training
- How was the training you received?
  - *Probe: in what ways did this provide you with the necessary skills? In what ways not?*
- Did you receive refresher training? How did this go? Which topics would you like to include in future refresher trainings?
- What could improve?

L. Tools
- What do you think about the tools you received to work with?
  - *Probe: take the tools and discuss*
- What is good?
- What should be changed?

M. Administration

- What do you think of the administration part of your job?
    - *Probe: workload, IT system,…*

N. Follow-up with supervisor

- How does the supervisor help you?
- Have you had the feeling you were supported and could share your worries, difficulties, challenges with the supervisor?
- What can be improved?

O. Incentives

- Do you feel like you are sufficiently reimbursed for the work that you do?
- Is this a valuable career experience for you?
    - *Probe: where do you see yourself after this?*

P. Future

- If the project could continue to exist. What should be kept the same?
- What should change?
    - *Do you have other ideas of what should be done differently?*
- If the project couldn't continue, what other ways do you see to share your experience and expertise?
    - *Probe: do you have specific organisations in mind?*

To end, imagine I was a new community health worker on my first day, what would you tell me that wasn't taught to you? What tips & trick would you give me?

- And what would you tell me to be mindful of?

PART 4: Concluding the focus group discussion

Thank you for participating in this focus group discussion.

Are there other things you would like to tell us?

Do you have any comments regarding this topic that you would like to share with us?

Do you have any questions for us?

<u>IN-DEPTH INTERVIEW (IDI) – SEMI-STRUCTURED INTERVIEW GUIDE RECIPIENTS CHW SUPPORT</u>

Getting to know the respondent

First, I would like to get to know you a little bit better. Could you briefly introduce yourself?
Probe: socio-demographic characteristics: age, migration-background etc

Thanks so much for your introduction.

Discussing barriers in access to care

Accessing care can be difficult for various reasons. Can I first ask you some questions about your relationship with care services in Belgium and the difficulties you encounter?

<u>Icebreaker: health of the participant</u>

- How is your health?
  - *Probe: last two weeks (acute)*
  - *Probe: chronic illnesses?*
- What do you do to maintain good health?

<u>Theme: barriers along the access to care continuum</u>

- When you are not feeling well, what do you do?
  - Where do you go to find information on what to do?
  - Who do you talk to for information or help when you are not feeling well?
    - *Probe: they go nowhere/do not do anything; just take a medicine that he/she bought previously or that a friend gives me to stop the pain (self-medication); he/she goes to the pharmacy*
  - If not going to the doctor: Why do you choose this option instead of going to the doctor?

- Do you ask for help from people in your neighbourhood (relatives, neighbours, associations) when you need healthcare?
  - What kind of help do you ask for (*Probe for: help to take an appointment, to fill administrative documents, to physically go to* your general practitioner/huisarts/….*)?*
  - What kind of help do you easily/not easily/never find?

- If you need care, where do you (or would you) go?
  - *Probe for: General Practitioner (huisarts), emergency care (spoed), community health centre (wijkgezondheidcentra)? Family? Community healer? No one?*
  - Why do you choose this type of care?
  - Have you ever gone to emergency care? What made you chose for this type of care?

- Do you have a personal GP?
  - Are there any other care services you meet regularly?
  - *Probe for dentist, nurse, specialists, home-services,…*

- When was the last time you went to the general practitioner/huisarts/…?
    - If long ago: What stops you from going again to that same/another general practitioner/huisarts/…?”.

- When you have been to the general practitioner/huisarts/…in the past, what has your experience been?
    - Was the general practitioner/huisarts/…able to help you?
        - If yes: How?
        - If no: Why not?
    - What challenges do you experience?

- What kind of insurance or support do you have to make sure you <u>are able to pay</u> for your care?
    - If the respondent does have an insurance:
        - In what way does this insurance help you to make sure you can pay for your healthcare?
        - Have you heard of other support options to help you to make sure you can pay for your healthcare?
    - If the respondent does not have an insurance: have you ever had an insurance?
        - If yes: What happened that you don’t have it anymore?
        - If no: Have you ever tried to get an insurance? What happened?

- Can you give an example of when you needed health care in Belgium and it was difficult?
    - Can you tell us a bit more about what happened?

- What could have helped you in that situation you just described to get the care you needed?

- What would be other things that could help you to overcome some of challenges we discussed today?

Discussing the CHW support received

Thank you for sharing all of this information with me, this is really useful. Let’s now briefly discuss the community health worker who has visited you.

<u>Theme: details of the CHW support</u>

- How many times have you met XXX (name of the CHW)?
- Where did you meet each other for the first time?
    - If met more than once: And after that?
- Why did XXX (name of the CHW) get in touch with you?
    - Are there other things XXX (name of the CHW) did not address?

<u>Theme: satisfaction with the support</u>

- How did XXXX (name of the CHW) help you?
- Are you happy with the support you receive from XXX (name of the CHW)?
- What do you like the most?

- What do you think could be improved?
- Are there things that you would like to see changed? What could be better?

Theme: the tools

- Did the CHW discuss any of these illustrations with you (show illustrations)?
  - If yes: what did you think about these? In which way did they help you?

Theme: Others

  - Were there other things that you had hoped the CHW could help you with?
  - Were there things that you did not like about the CHW's support? (*Probe for unintended consequences of the CHW's support)*

Concluding the IDI

Thank you for participating in this Interview.

- Are there other things you would like to tell us?
- Do you have any comments regarding this topic that you would like to share with us?
- Do you have any questions for us?
